# Associations between comorbidities and annual incidence plus frequency of asthma exacerbation hospitalisation during the past year: data from CARN study

**DOI:** 10.1186/s12890-022-02038-3

**Published:** 2022-07-01

**Authors:** Wenqiao Wang, Jiangtao Lin, Xin Zhou, Changzheng Wang, Mao Huang, Shaoxi Cai, Ping Chen, Qichang Lin, Jianying Zhou, Yuhai Gu, Yadong Yuan, Dejun Sun, Xiaohong Yang, Lan Yang, Jianmin Huo, Zhuochang Chen, Ping Jiang, Jie Zhang, Xianwei Ye, Huiguo Liu, Huaping Tang, Rongyu Liu, Chuntao Liu, Wei Zhang, Chengping Hu, Yiqiang Chen, Xiaoju Liu, Luming Dai, Wei Zhou, Yijiang Huang, Jianying Xu

**Affiliations:** 1 Department of Pulmonary and Critical Care Medicine, China-Japan Friendship Hospital, Beijing, 100029 China; 2 Department of Respiration, Shanghai Central Hospital, Shanghai, China; 3 Department of Respiration, Xinqiao Hospital, Third Military Medical University, Chongqing, China; 4 Department of Pulmonary and Critical Care Medicine, The First Affiliated Hospital of Nanjing Medical University, Nanjing, China; 5 Department of Respiration, Nanfang Hospital, Guangzhou, China; 6 Department of Respiratory Diseases, General Hospital of Shenyang Military Command, Shenyang, China; 7 Department of Respiration, The First Affiliated Hospital of Fujian Medical University, Fuzhou, China; 8 Department of Respiration, The First Affiliated Hospital of Zhejiang University School of Medicine, Hangzhou, China; 9 Department of Respiration, Qinghai People’s Hospital, Xining, China; 10 Department of Respiration, The Second Hospital of Hebei Medical University, Shijiazhuang, China; 11 Department of Pulmonary and Critical Care Medicine, Inner Mongolia People’s Hospital, Hohhot, China; 12 Department of Pulmonary and Critical Care Medicine, People’s Hospital of Xinjiang Uygur Autonomous Region, Urumqi, China; 13 Department of Pulmonary and Critical Care Medicine, The First Affiliated Hospital of Xi’an Jiaotong University, Xi’an, China; 14 Department of Respiration, The First Affiliated Hospital of Harbin Medical University, Harbin, China; 15 Department of Respiration, Henan Provincial People’s Hospital, Zhengzhou, China; 16 Department of Respiration, Tianjin First Central Hospital, Tianjin, China; 17 Department of Pulmonary and Critical Care Medicine, The Second Hospital of Jilin University, Changchun, China; 18 Department of Respiration, Guizhou Provincial People’s Hospital, Guiyang, China; 19 Department of Respiration, Tongji Hospital, Wuhan, China; 20 Department of Respiration, Qingdao Municipal Hospital, Qingdao, Shandong China; 21 Department of Respiration, The First Affiliated Hospital of Anhui Medical University, Hefei, China; 22 Department of Respiratory and Critical Care Medicine, West China School of Medicine and West China Hospital, Sichuan University, Chengdu, China; 23 Department of Respiration, The First Affiliated Hospital of Nanchang University, Nanchang, China; 24 Department of Respiration, Xiangya Hospital, Changsha, China; 25 Department of Respiration, The First Affiliated Hospital of Guangxi Medical University, Nanning, China; 26 Department of Respiration, The First Affiliated Hospital of Lanzhou University, Lanzhou, China; 27 Department of Respiration, Kunming General Hospital of the People’s Liberation Army, Kunming, China; 28 Department of Respiration, General Hospital of Ningxia Medical University, Yinchuan, China; 29 Department of Respiration, Hainan General Hospital, Haikou, China; 30 Department of Respiration, Shanxi Bethune Hospital, Taiyuan, China

**Keywords:** Asthma, Exacerbation, Hospitalisation, Comorbidity, Multi-centre cross-sectional study

## Abstract

**Purpose:**

While asthma comorbidities are associated with higher health care utilisation, lower quality of life and poorer asthma control, the impact of asthma comorbidities on hospitalisation for asthma exacerbation (H-AX) remains less recognised. We aim to analyse the impact of asthma comorbidities on H-AX.

**Methods:**

Based on a national survey on asthma control and disease perception (CARN 2015 study), we analysed the impact of comorbidities on annual incidence and frequency of H-AX in China. Information on demographic characteristics, asthma comorbidities and annual incidence and frequency of H-AX were presented in this study.

**Results:**

Among 3875 ambulatory asthma patients, 75.9% (2941/3875) had comorbidities, and 26.4% (1017/3858) experienced H-AX during past year. After adjusting for confounding factors such as demographic data, smoking status and asthma control, COPD [OR = 2.189, 95% CI (1.673, 2.863)] and coronary heart disease [OR = 1.387, 95% CI (1.032, 1.864)] were associated with higher annual incidence, while allergic rhinitis [OR = 0.692, 95% CI (0.588, 0.815)] was associated with lower annual incidence, of H-AX. In terms of frequency, allergic rhinitis [OR = 1.630, 95% CI (1.214, 2.187)], COPD [OR = 1.472, 95% CI (1.021, 2.122)] and anxiety [OR = 2.609, 95% CI (1.051, 6.477)] showed statistically significant correlation with frequent H-AX.

**Conclusions:**

COPD and coronary heart disease were associated with higher annual incidence, while allergic rhinitis was associated with lower annual incidence of H-AX. Allergic rhinitis, COPD and anxiety were associated with frequent H-AX. Comorbidities may have an important role in the risk and frequency of annual hospitalisations due to asthma exacerbation. The goal of asthma control should rely on a multi-disciplinary treatment protocol.

## Introduction

Bronchial asthma is a heterogeneous disease characterised by chronic airway inflammation and associated with hefty social and economic burdens [1]. Asthma exacerbations, especially those necessitating hospital admissions, contribute to the majority of the healthcare expenses in asthma patients [2]. The risk factors of asthma exacerbation generally include viral infection of the upper respiratory tract [3], exposure to allergens (such as grass pollens [4], soy bean dusts [5], fungal spores, and food allergens [6]), outdoor and indoor air pollution [7–10], occupational exposure [11, 12], climatic seasonality [13] and poor adherence with inhaled corticosteroids (ICS) therapy [14].

Besides these, having uncontrolled asthma is inherently related to frequent exacerbations [15]. The importance of asthma assessment and management therefore cannot be under-estimated. Reducing asthma exacerbation was an important asthma management goal [1]. Though several studies have shown that comorbidities are associated with higher health care utilisation, lower quality of life and poorer asthma control [16–19], respiratory physicians still put more emphasis on asthma treatment, avoidance of allergen exposure, but paid less attention on comorbidities management in clinical management of asthma, which indicated the role of comorbidities on asthma exacerbations remains less recognised. In order to investigate the role of comorbidities on hospitalisation for asthma exacerbation (H-AX) in a huge population of Chinese patients, we analysed the association between comorbidities and annual incidence & frequency of asthma exacerbation hospitalisation during the previous year using data from a national survey on asthma control and disease perception.

## Materials and methods

### Study design and participants

This study was based on a national multi-centre, cross-sectional, questionnaire-based survey on asthma control and disease perception conducted by China Asthma Research Network (CARN 2015 study) [20]. Briefly, the CARN 2015 study was a multi-centre, cross-sectional, questionnaire-based survey, carried out in 30 provinces of China from October 2015 to May 2016. We used stratified rondomization and aimed to recruited 150 cases in each province. Due to some practical reasons, one province in mainland China (Tibet) was not included in this survey. One hospital was chosen in each province. In each center, asthma outpatients who met all the including criteria were included sequentially in outpatient department of respiratory department. Altogether 3875 asthma patients from 30 centres were recruited who met all of the following: (1) age ≥ 14 years old; (2) having resided in the study city for at least 2 years; (3) diagnosed with asthma at least 3 months prior to the study according to GINA criteria. In that study steered with approval by the Ethics Committee of China Japan Friendship Hospital, data on demography, asthma control, medical and self-management, exacerbations, and disease perception were collected during face-to-face interviews with written informed consent from the patients. In the first section of questionnaire on demography, data of comorbidities was collected based on a multiple-choice question on comorbidities with supplement if there were other comorbidities beyond the choices. In the second section of questionnaire on asthma control, data of hospitalizations due to asthma exacerbation was collected based on two questions: (1) whether or not experienced asthma exacerbation hospitalization during the past year; (2) asthma exacerbation hospitalization frequency during the past year. All interviews were conducted with assistance with respiratory physicians in outpatient department in each centre for part of the questions were too professional for outpatients. Each interview took 20–30 min.

Recently, to shed light on the impacts of comorbidities on annual incidence and frequency of H-AX, we extracted the following data from all participants in the CARN 2015 study: (1) Demographic characteristics including age, gender, height, weight, and body mass index (BMI); (2) Self-reported comorbidities, including atopic diseases (allergic rhinitis, nasosinusitis, rhinopolypus, and food allergy), respiratory diseases [chronic obstructive pulmonary disease (COPD), bronchiectasis, and obstructive sleep apnea hypopnea syndrome (OSAHS)], cardiovascular diseases (hypertension, coronary heart disease), metabolic disorders (obesity and diabetes), digestive conditions [gastroesophageal reflux disease (GERD)], cerebrovascular disease, psychiatric disorders (depression and anxiety), and other comorbidities such as osteoporosis; (3) Annual incidence and frequency of H-AX during the year prior to CARN 2015. The data extraction, database input and double-checking were completed by a designated investigator (WQW) under the supervision of our team leader (JTL). Use of CARN 2015 data in the present study was again approved by the Ethics Committee of China Japan Friendship Hospital (No. 2015-98) with the request to protect patient identity and privacy.

According to GINA 2021, asthma exacerbations were defined as episodes characterised by a progressive increase in symptoms of shortness of breath, cough, wheezing or chest tightness and progressive decline in lung function, which represent a change from the patient’s usual status sufficiently to require a change in treatment [1]. H-AX was defined as any hospitalisation due to asthma exacerbations. According to H-AX in the previous year, the patients who had experienced H-AX were assigned to the H-AX group, and those who had not, into the non-H-AX group. The H-AX group was further stratified into three sub-groups, where the frequency of previous-year H-AX was one, two or at least three, respectively.

### Statistical analysis

Continuous variables (age, height, weight, and BMI) were presented as mean ± SD and categorical variables (gender, comorbidities) were presented as frequency. Between-group comparisons were completed using unpaired t test for continuous variables and Chi-square test for categorical variables. Levene test was made for homoscedasticity check prior to t test. Binary logistic regression, followed by multivariate logistic regression, was performed to assess the effect of comorbidities on the incidence of H-AX after adjusting for confounders (demographic status, smoking status, and asthma control). Collinearity test was conducted prior to regression model. Height, weight, BMI was not included in regression model for high collinearity. All statistics adopted fixed α significance level to 0.05. All data were processed with SPSS statistical software (version 21.0; IBM SPSS, Armonk, NY, USA).

## Results

### Demographics and comorbidities

Data retrieved from the CARN 2015 study with 3875 ambulatory asthma patients, including 2347 females (60.6%), were available for the present study. The mean age was 50.7 ± 16.7 years. Of these patients, 75.9% (2941/3875) had comorbidities. Specifically, 43.4% (1682/3875) had allergic rhinitis, 16.4% (634/3875) had hypertension, 8.7% (338/3875) had nasosinusitis, 7.3% (283/3875) had COPD, and 3.0% (118/3875) had bronchiectasis. Asthma control was achieved in 28.5% (1099/3854) of the patients. The demographic characteristics is shown in Table 1.

**Table 1:** Demographics and comorbidities

Items	Mean ± SD	Percentage [% (n/N)]
Gender		
Male		39.4% (1528/3875)
Female		60.6% (2347/3875)
Age (years)	50.7 ± 16.7	
Height (cm)	163.8 ± 7.9	
Weight (kg)	63.8 ± 12.4	
BMI (kg/m^2^)	23.7 ± 3.9	
Comorbidities		
Allergic rhinitis		43.4% (1682/3875)
Nasosinusitis		8.7% (338/3875)
Rhinopolypus		2.9% (114/3875)
Food allergy		6.0% (232/3875)
COPD		7.3% (283/3875)
Bronchiectasis		3.0% (118/3875)
OSAHS		2.9% (114/3875)
Hypertension		16.4% (634/3875)
CHD		6.6% (256/3875)
Obesity		1.5% (58/3875)
Diabetes		4.9% (188/3875)
GERD		3.7% (143/3875)
CVD		6.6% (256/3875)
Depression		0.6% (22/3875)
Anxiety		1.6% (63/3875)
Osteoporosis		1.6% (63/3875)
Others		10.6% (409/3875)

Data are presented as mean ± standard deviations (SD) or percentage (%, n/N)

BMI, body mass index; COPD, chronic obstructive pulmonary disease; OSAHS, obstructive sleep apnea–hypopnea syndrome; CHD, Coronary heart disease; GERD, gastroesophageal reflux disease; CVD, Cerebrovascular disease

**Fig. 1:**
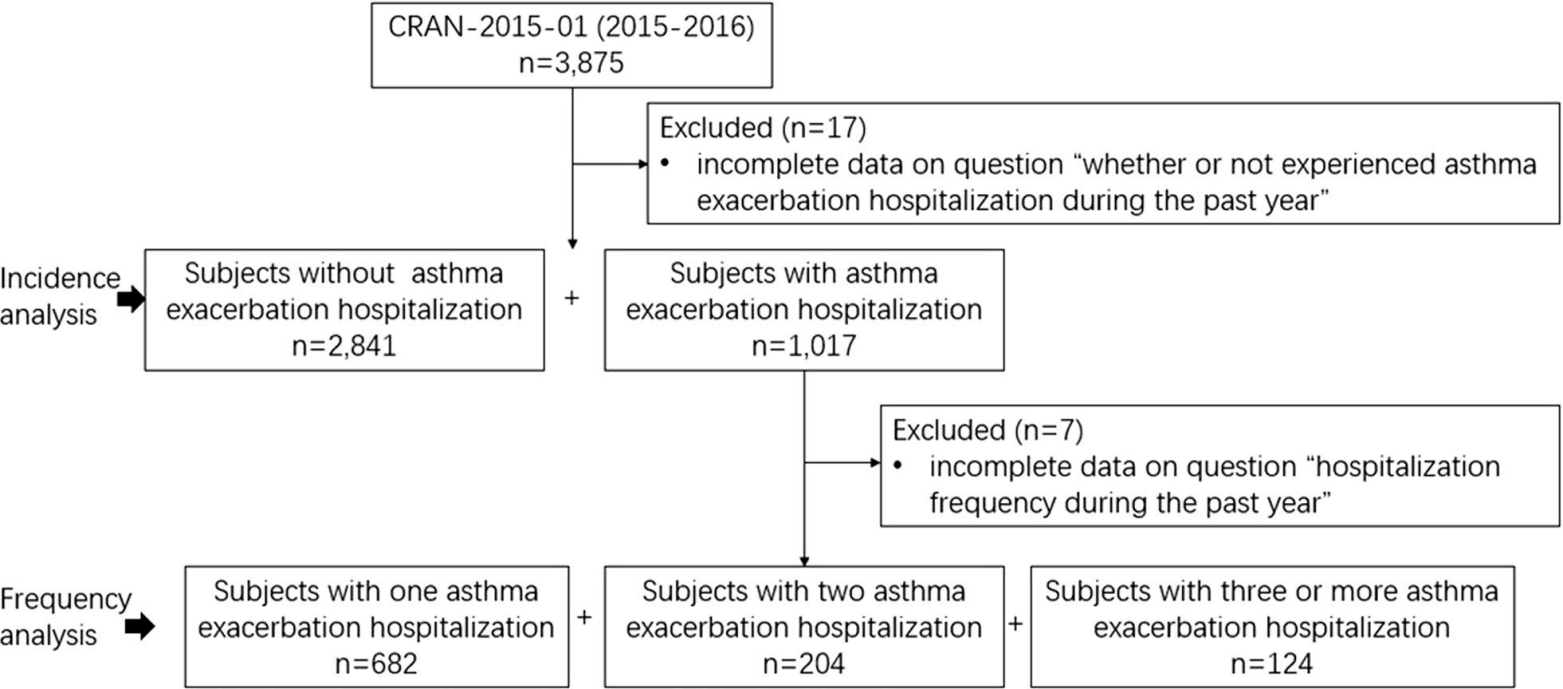
Flow chart of the present study

To elucidate on the impacts of comorbidities on the incidence and frequency of H-AX, 17 patients with missing report on the previous-year hospitalisation due to asthma, and likewise, seven more who reported so but did not specify the number of H-AX were excluded from the subsequent analyses (Fig. 1).

### Associations between comorbidities and annual incidence of H-AX

Of the study population, 26.4% (1017/3858) of the study population were hospitalised due to asthma exacerbation during the previous year. Compared to those without H-AX, the asthma patients in the H-AX group were more likely to have advanced age (57.5 ± 14.9 vs. 48.6 ± 15.7 years, P < 0.001) and lower height (163.1 ± 7.7 vs. 164.1 ± 8.0 cm, P < 0.001). With regards to comorbidities, except for the lower proportion of allergic rhinitis (32.0% vs. 47.4%, χ^2^ = 73.179, P < 0.001), patients with previous-year H-AX were more likely to have concomitant COPD (15.4% vs. 5.5%, χ^2^ = 133.375, P < 0.001), bronchiectasis (4.6% vs. 2.5%, χ^2^ = 11.377, P = 0.001), hypertension (23.3% vs. 13.9%, χ^2^ = 48.736, P < 0.001), coronary heart disease (11.4% vs. 4.9%, χ^2^ = 52.220, P < 0.001), and diabetes (8.2% vs. 3.6%, χ^2^ = 33.577, P < 0.001). The proportions of other comorbidities did not differ statistically between the two groups (Table 2).

**Table 2:** Subjects with and without the previous-year H-AX

Items	H-AX group	Non-HAX group	t value	χ^2^ value	P value
Gender					
Male	41.6% (423/1017)	38.6% (1098/2841)		2.719	0.099
Female	58.4% (594/1017)	61.4% (1743/2841)			
Age (years)	57.5 ± 14.9	48.6 ± 15.7	− 16.149		< 0.001*
Height (cm)	163.1 ± 7.7	164.1 ± 8.0	3.562		< 0.001*
Weight (kg)	63.3 ± 12.2	64.1 ± 12.5	1.715		0.086
BMI (kg/m^2^)	23.7 ± 3.8	23.7 ± 3.0	− 0.012		0.990
Comorbidities					
Allergic rhinitis	32.0% (325/1017)	47.4% (1348/2841)		73.179	< 0.001*
Nasosinusitis	8.2% (83/1017)	9.0% (255/2841)		0.621	0.430
Rhinopolypus	2.8% (28/1017)	3.0% (86/2841)		0.196	0.658
Food allergy	6.2% (63/1017)	5.9% (167/2841)		0.134	0.715
COPD	15.4% (157/1017)	5.5% (157/2841)		133.375	< 0.001*
Bronchiectasis	4.6% (47/1017)	2.5% (71/2841)		11.377	0.001*
OSAHS	3.0% (31/1017)	2.9% (83/2841)		0.042	0.838
Hypertension	23.3% (237/1017)	13.9% (394/2841)		48.736	< 0.001*
CHD	11.4% (116/1017)	4.9% (138/2841)		52.220	< 0.001*
Obesity	1.7% (17/1017)	1.4% (41/2841)		0.264	0.607
Diabetes	8.2% (83/1017)	3.6% (103/2841)		33.577	< 0.001*
GERD	3.2% (33/1017)	3.9% (110/2841)		0.825	0.364
CVD	1.3% (13/1017)	1.5% (44/2841)		0.376	0.540
Depression	0.5% (5/1017)	0.6% (17/2841)		0.150	0.698
Anxiety	1.9% (19/1017)	1.5% (44/2841)		0.476	0.490
Osteoporosis	2.1% (21/1017)	1.5% (42/2841)		1.604	0.205
Others	11.7% (119/1017)	10.1% (288/2841)		1.941	0.164

Data are presented as mean ± standard deviation (SD) or percentage (%, n/N)

H-AX, Hospitalisation due to asthma exacerbation; BMI, body mass index; COPD, chronic obstructive pulmonary disease; OSAHS, obstructive sleep apnea–hypopnea syndrome; CHD, Coronary heart disease; GERD, gastroesophageal reflux disease, CVD, Cerebrovascular disease

*Data with statistical significance

After adjusting for confounding factors such as demographic data, smoking status and asthma control, COPD [OR = 2.189, 95% CI (1.673, 2.863)] and coronary heart disease [OR = 1.387, 95% CI (1.032, 1.864)) were associated with higher annual incidence, while allergic rhinitis [OR = 0.692, 95% CI (0.588, 0.815)] was associated with lower annual incidence, of H-AX (Table 3).

**Table 3:** Associations between comorbidities and incidence of H-AX during the past year

Comorbidities	OR	95% CI	P value
Allergic rhinitis	0.692	(0.588, 0.815)	< 0.001*
Nasosinusitis	1.116	(0.839, 1.485)	0.449
Rhinopolypus	1.065	(0.664, 1.708)	0.794
Food allergy	1.282	(0.928, 1.773)	0.132
COPD	2.189	(1.673, 2.863)	< 0.001*
Bronchiectasis	1.379	(0.921, 2.066)	0.119
OSAHS	0.953	(0.607, 1.497)	0.834
Hypertension	1.107	(0.894, 1.369)	0.351
CHD	1.387	(1.032, 1.864)	0.030*
Obesity	1.079	(0.581, 2.004)	0.809
Diabetes	1.284	(0.922, 1.787)	0.140
GERD	0.776	(0.509, 1.183)	0.239
CVD	0.399	(0.203, 0.783)	0.008*
Depression	0.887	(0.300, 2.623)	0.828
Anxiety	1.076	(0.599, 1.935)	0.805
Osteoporosis	1.039	(0.584, 1.850)	0.896

Data are presented as mean ± standard deviation (SD) or percentage (n/N)

OR, Odds ratio; CI, Confidence interval; H-AX, Hospitalisation due to asthma exacerbation; BMI, body mass index; COPD, chronic obstructive pulmonary disease; OSAHS, obstructive sleep apnea–hypopnea syndrome; CHD, Coronary heart disease; GERD, gastroesophageal reflux disease; CVD, Cerebrovascular disease

Collinearity test was conducted prior to regression test. The VIF value of height, weight and BMI was greater than 10, which indicated high collinearity. Thus height, weight and BMI was not included in regression model. Other confounding factors which passed collinearity test (gender, age, smoking history, asthma control level) were included in regression model

*Data with statistical significance

We made further analysis on number of comorbidities and annual incidence of hospital admissions for asthma. As the number of comorbidities increased, the annual incidence of H-AX increased: 24.1%(273/1131) for patients with no comorbidities, 25.2%(425/1685) for patients with one comorbidity, 27.9%(181/648) for patients with two comorbidities, 35.4% (92/260) patients with three comorbidities, 34.5%(30/87) for patients with four comorbidities, 25.0%(8/32) for patients with five comorbidities, 41.7%(5/12) for patients with six comorbidities, 100.0% (2/2) for patients with seven comorbidities and 100.0% (1/1) for patients with twelve comorbidities. χ^2^ value was 28.550, P < 0.001, which indicated the difference of annual incidence of H-AX in groups with different number of comorbidities was statistically significant.

**Table 4:** Associations between comorbidities and frequent H-AX during the past year

Comorbidities	OR	95% CI	P value
Allergic rhinitis	1.630	(1.214, 2.187)	0.001*
Nasosinusitis	1.566	(0.964, 2.544)	0.070
Rhinopolypus	1.108	(0.498, 2.466)	0.801
Food allergy	0.807	(0.454, 1.435)	0.465
COPD	1.472	(1.021, 2.122)	0.038*
Bronchiectasis	1.173	(0.637, 2.158)	0.609
OSAHS	0.901	(0.406, 2.000)	0.799
Hypertension	0.994	(0.702, 1.407)	0.972
CHD	1.026	(0.660, 1.595)	0.910
Obesity	0.822	(0.278, 2.435)	0.724
Diabetes	1.557	(0.961, 2.522)	0.072
GERD	1.538	(0.762, 3.106)	0.230
CVD	2.173	(0.721, 6.552)	0.168
Depression	0.304	(0.029, 3.225)	0.323
Anxiety	2.609	(1.051, 6.477)	0.039*
Osteoporosis	1.200	(0.477, 3.019)	0.699

Data are presented as mean ± standard deviation (SD) or percentage (n/N)

OR, Odds ratio; CI, Confidence interval H-AX, Hospitalisation due to asthma exacerbation; BMI, body mass index; COPD, chronic obstructive pulmonary disease; CHD, Coronary heart disease; OSAHS, obstructive sleep apnea–hypopnea syndrome; GERD, gastroesophageal reflux disease; CVD, Cerebrovascular disease

*Data with statistical significance

### Associations between comorbidities and frequency of the previous-year H-AX

**Fig. 2:**
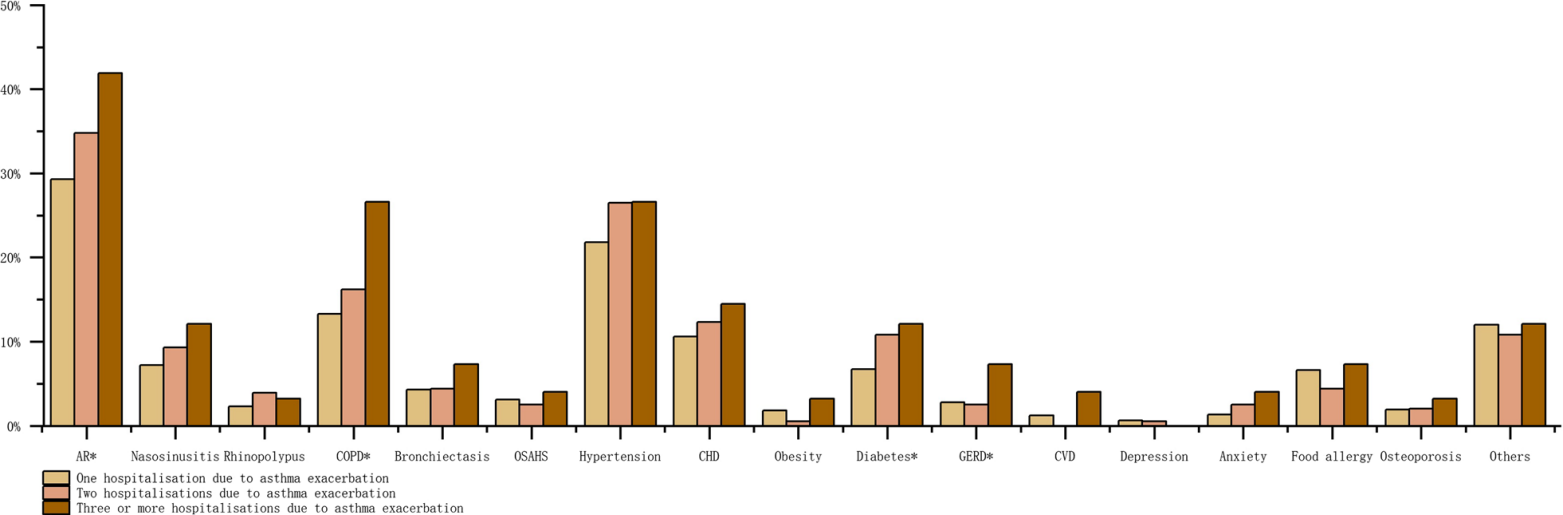
Comorbidities in subgroup with different H-AX frequency during the past year. Data are presented as percentage. *Data with statistical significance. AR, allergic rhinitis; COPD, chronic obstructive pulmonary disease; OSAHS, obstructive sleep apnea–hypopnea syndrome; CHD, Coronary heart disease; GERD, gastroesophageal reflux disease; CVD, Cerebrovascular disease

Frequency of H-AX during the previous year was reported by 1010 asthma patients. Among them, 67.5% (682/1010) experienced one H-AX, 20.2% (204/1010) experienced two, and 12.3% (124/1010) experienced three or more during the year prior to CARN 2015 study. The asthma patients with frequent H-AX (two, three or more H-AX) tended to be more affected by comorbidities, in particular, by allergic rhinitis (P = 0.014), COPD (P = 0.001), diabetes (P = 0.045), GERD (P = 0.028), and cerebrovascular diseases (P = 0.006). Data were shown in Fig. 2.

After adjusting for confounding factors such as demographic data, smoking status and asthma control level, allergic rhinitis [OR = 1.630, 95% CI (1.214, 2.187)], COPD [OR = 1.472, 95% CI (1.021, 2.122)] and anxiety [OR = 2.609, 95% CI (1.051, 6.477)] were significant associated with frequent asthma exacerbation hospitalisation (two, three or more H-AX) (Table 4).

## Discussion

In this study, we investigated the association between comorbidities and hospitalisation for asthma exacerbation (H-AX) based on CARN 2015, a multi-centre cross-sectional survey participated by 3875 asthma patients in China. As shown, 75.9% of our study population suffered a wide range of comorbidities involving the lungs, heart, vessels, immunity and metabolism. Over a quarter (26.4%) experienced at least one hospitalisation for asthma exacerbation during the previous year, of whom, 12.3% experienced three or more hospitalisations. COPD and coronary heart disease were associated with higher annual incidence, while allergic rhinitis was associated with lower annual incidence of H-AX. Allergic rhinitis, COPD and anxiety were associated with frequent H-AX. The results indicated comorbidities in asthma patients played an important role in H-AX events.

Of allergic comorbidities, nasosinusitis and rhinopolypus are common in asthma patients, and were found in 75–80% of severe asthma cases [21, 22]. Nasosinusitis and confirmed food allergy are independent risk factors of asthma exacerbation [6, 23]. Several previous studies indicated poorer asthma outcome with comorbidity of allergic rhinitis [24–26]. In this study, we failed to determine association between nasosinusitis, rhinopolypus, food allergy, and asthma exacerbation or frequent H-AX. Surprisingly, asthma patients who experienced H-AX presented lower prevalence of allergic rhinitis; we speculated that this puzzling observation may be associated the higher rate of co-treatment in patients with mild allergic rhinitis, although we did not perform a subgroup analysis for demonstration. However, in terms of H-AX frequency, allergic rhinitis was associated with two or more hospitalisation related to asthma in the previous year, which indicated allergic rhinitis might be a risk factor to frequent asthma exacerbation hospitalizations.

Cardiovascular disease can influence asthma outcomes, and vice versa. Schanen et al. [27] reported that asthma patients were more likely to have cardiovascular disease. In a previous survey among adults aged ≥ 65 years, asthma patients with coronary artery disorders showed fairly higher adjusted odd ratios for one or more asthma-related hospitalisations [28]. In the present study, cardiovascular comorbidities were linked to H-AX. We indicated that coronary heart disease was associated with significantly higher annual incidence, but not frequency, of H-AX.

Metabolic disorders are also common in asthma. Obesity has been reported to relates with increased asthma severity and exacerbations [29]. In this setting, data about diabetes remain limited. Song et al. found that women who had ever reported asthma or COPD were at a higher risk for diabetes [30, 31]. Diabetes and insulin resistance are associated with decline in lung function [32–34]. In our study, having diabetes was associated with significantly higher incidence and frequency of H-AX in univariate analyses, but the statistical differences were not reached in subsequent multivariate logistic analyses. In either univariate or multivariate analysis, cormorbidity with obesity did not correlate with H-AX in incidence or frequency. Therefore, the role of metabolic disorders in H-AX warrants future studies.

The interplay between asthma and respiratory comorbidities, structural lung diseases in particular, should be noteworthy to mention. Compared to asthma or COPD alone, asthma-COPD overlap leads to heavier burden of symptoms [35], incurs more frequent exacerbations [35–37] and accounts for greater use of healthcare resources [36, 38]. Mao et al. [39] noted that concomitant asthma was associated independently with an increase in risk of bronchiectasis exacerbation. In contrast, few studies assessed the impact of bronchiectasis on asthma exacerbation. Kang et al. showed higher annual incidence of asthma exacerbation and frequency of emergency room visits in patients with asthma and bronchiectasis than in those with asthma alone [40]. In the present study, we demonstrated that comorbidity with structural lung diseases, such as COPD, was associated with both higher annual incidence and frequency of H-AX. We believe that our findings add to the evidence supporting the adverse impacts of structural lung disease on asthma outcome.

Concomitant GERD is estimated to affect 34–89% of asthma patients [41], and has been linked to the severity of asthma [42, 43]. However, our results indicated neither a higher prevalence of GERD in the asthma patients, nor a statistical association of GERD with the likelihood of H-AX. We speculated the self-reporting of comorbidities in CARN 2015 study might undercut the GERD prevalence. Notwithstanding this, in our study, GERD was associated with more frequent H-AX in the univariate analysis albeit with no statistical significance in the multivariate logistic regression.

Finally, we need to elaborate on several special considerations pertaining to the strength and weakness of our study. Firstly, to the best of our knowledge and data availability, this study is so far the largest nationwide survey on H-AX and comorbidities among Chinese asthmatics over the recent years. Using relevant information from full dataset of CARN 2015, our findings regarding the relationship between certain comorbidities and the incidence and/or frequency of H-AX in asthma patients could therefore be a close reflection of the real world in China. Secondly, comorbidities associated to the risk and/or frequency of H-AX in this study chiefly involved structural lung diseases and chronic, systemic disorders. Given that this was merely an observational rather than a mechanistic study, our findings should be interpreted with prudence and do not mean to propose a causative relationship. Nevertheless, comorbid abnormality in pulmonary architecture, systemic inflammation and immune function, could impose unfavorable impacts on the natural history and treatment outcomes of asthma which is an immune, inflammatory disorder per se. In this context, the goal of asthma control can be achieved not only depending on efforts of respiratory physicians, but also involving a multi-disciplinary treatment protocol. Either other systemic conditions as comorbidity in asthma, or asthma as comorbidity in other systemic conditions, need to be ideally taken together in decision-making for treatments. Thirdly, criteria have not been widely recognised for “frequent” asthma exacerbations or “frequent” H-AX. As an attempt for description in this study, we tentatively stratified the asthma patients according to one, two, three or more H-AX in the previous year and also by taking into consideration the comorbidities. The times of H-AX statistically differed with such stratification. Our statistics showed that using annual sessions ≥ 2 or ≥ 3 in a patient was comparably acceptable to define exacerbations as “frequent” in asthma. In this regard, our work may add to determination of the definition of frequent asthma exacerbation, rendering more discussions needed in the future.

The study has several limitations. Self-reported comorbidities may underestimate the real prevalence and be influenced by interviewees compliance. More confounding factors, such as medications and treatment adherence, should have been included in analyses. Future investigations with follow-up study or comorbidity intervention study would help to validate our findings and clarify more on the relationship between comorbidities and hospitalisation due to exacerbation in asthma patients.

## Conclusion

COPD and coronary heart disease were associated with higher annual incidence, while allergic rhinitis was associated with lower annual incidence of H-AX. Allergic rhinitis, COPD and anxiety were associated with frequent H-AX. Comorbidities may have an important role in the risk and frequency of annual hospitalisations due to asthma exacerbation. The goal of asthma control should rely on a multi-disciplinary treatment protocol.

## Data Availability

The datasets are available from the corresponding author on reasonable request.
